# The RL13 Temperance Factor Represses Replication of the Highly Cell Culture-Adapted Towne Strain of Human Cytomegalovirus

**DOI:** 10.3390/v15041023

**Published:** 2023-04-21

**Authors:** Amine Ourahmane, Laura Hertel, Michael A. McVoy

**Affiliations:** 1 Department of Microbiology & Immunology, Virginia Commonwealth University, Richmond, VA 23298, USA; ourahmanea@vcu.edu; 2 Department of Pediatrics, University of California San Francisco, Oakland, CA 94609, USA; laura.hertel@ucsf.edu; 3 Department of Pediatrics, Virginia Commonwealth University, Richmond, VA 23298, USA

**Keywords:** cytomegalovirus, RL13, virion assembly compartment, temperance factor, adaptive mutations

## Abstract

Human cytomegalovirus (CMV) has evolved to replicate while causing minimal damage, maintain life-long latency, reactivate sub-clinically, and, in spite of robust host immunity, produce and shed infectious virus in order to transmit to new hosts. The CMV temperance factor RL13 may contribute to this strategy of coexistence with the host by actively restricting viral replication and spread. Viruses with an intact *RL13* gene grow slowly in cell culture, release little extracellular virus, and form small foci. By contrast, viruses carrying disruptive mutations in the *RL13* gene form larger foci and release higher amounts of cell-free infectious virions. Such mutations invariably arise during cell culture passage of clinical isolates and are consistently found in highly adapted strains. The potential existence in such strains of other mutations with roles in mitigating RL13’s restrictive effects, however, has not been explored. To this end, a mutation that frame shifts the *RL13* gene in the highly cell culture-adapted laboratory strain Towne was repaired, and a C-terminal FLAG epitope was added. Compared to the frame-shifted parental virus, viruses encoding wild-type or FLAG-tagged wild-type RL13 produced small foci and replicated poorly. Within six to ten cell culture passages, mutations emerged in *RL13* that restored replication and focus size to those of the *RL13*-frame-shifted parental virus, implying that none of the numerous adaptive mutations acquired by strain Towne during more than 125 cell culture passages mitigate the temperance activity of RL13. Whilst RL13-FLAG expressed by passage zero stocks was localized exclusively within the virion assembly compartment, RL13-FLAG with a E208K substitution that emerged in one lineage was mostly dispersed into the cytoplasm, suggesting that localization to the virion assembly compartment is likely required for RL13 to exert its growth-restricting activities. Changes in localization also provided a convenient way to assess the emergence of *RL13* mutations during serial passage, highlighting the usefulness of RL13-FLAG Towne variants for elucidating the mechanisms underlying RL13’s temperance functions.

## 1. Introduction

Human cytomegalovirus (CMV) is a herpesvirus that infects the majority of the human population. In immunocompetent subjects, primary infections are mild or asymptomatic but result in the establishment of life-long latency. Periodic reactivations from latency are asymptomatic and serve to maintain robust CMV-specific humoral and cellular immunity. As a consequence, CMV has evolved to replicate while causing minimal damage, maintain life-long latency, reactivate sub-clinically, and, in spite of robust host immunity, produce and shed infectious virus in order to transmit to new hosts.

In the absence of functional host immunity, however, both primary infections and reactivations cause a spectrum of diseases, including retinitis in AIDS patients and pneumonitis in transplant patients. CMV can also be transmitted transplacentally, resulting in fetal infections that can cause serious birth defects, including sensorineural hearing loss and severe mental retardation. Options for preventing or treating CMV infections are limited as no vaccine is available, and existing antivirals are hampered by dose-limiting toxicities and the development of resistance. These limitations have fostered continued efforts to understand the molecular biology and natural history of CMV infections to inform the development of effective vaccines and to identify novel targets for antiviral interventions. 

In contrast to many viral pathogens that cause acute disease associated with rapid viral replication, CMV encodes temperance factors that actively restrict its replication and spread [1]. One such temperance factor, RL13, causes CMV to replicate slowly, release little cell-free infectious virus, and form small plaques in monolayer cultures [2,3]. Consequently, cell culture passage imposes strong selective pressures favoring the rapid emergence of mutations in the *RL13* gene with ensuing enlargement of focus size and 3–4 log_10_ increases in cell-free infectious progeny [2,3,4]. 

RL13 is an envelope-associated type I integral membrane glycoprotein with conserved endo and transmembrane domains and a highly polymorphic ectodomain [2]. The primary translation product of *RL13* is predicted to be a 35-kDa protein comprised of a signal sequence, transmembrane domain, seven potential N-linked glycosylation sites, and 26 potential O-linked glycosylation sites [5]. Previous work on RL13 has been mainly conducted in the context of clinical strains lacking adaptive mutations that arise during extended cell culture passage. Consequently, it is not known whether such adaptive mutations might function to mitigate the temperance activities of RL13. 

In the present work, a mutation that frame shifts the *RL13* gene in the highly cell culture-adapted laboratory strain Towne was repaired, and a C-terminal FLAG epitope was added. The resulting viruses produced small foci, replicated poorly, and rapidly acquired *RL13* mutations, indicating that RL13 is active as a temperance factor in the Towne strain background and that its activity is not impaired by the C-terminal FLAG epitope. Wild-type RL13-FLAG was exclusively localized in the virion assembly compartment (VAC), while RL13-FLAG encoded by stocks that had acquired a single amino acid substitution in *RL13* was mostly dispersed into the cytoplasm.

Together, these data suggest that none of the adaptive mutations carried by the highly passaged Towne strain antagonize RL13’s temperance activities and that such functions likely require accumulation of RL13 in the VAC, as RL13 mis-localization into the cytoplasm appears to attenuate its negative effects on virion assembly and/or egress.

## 2. Materials and Methods

### 2.1. Cells

Human MRC-5 fetal lung fibroblasts (ATCC CCL-171) were obtained from American Type Culture Collection. Human foreskin fibroblast (HFF) cells were a gift from E. S. Mocarski, Emory University, Atlanta, GA. All cells were cultivated in high glucose Dulbecco’s modified Eagle medium (Life Technologies) supplemented with 10% heat-inactivated fetal bovine serum (FBS) (Life Technologies), 10,000 IU/L penicillin, 10-mg/L streptomycin sulfate, and 29.2 mg/mL L-glutamine (Life Technologies) (culture medium).

### 2.2. Viruses

Recombinant viruses were derived by genetic modification of the TL12 bacterial artificial chromosome (BAC) clone of the CMV strain Towne-varL genome modified to encode green fluorescent protein (GFP) [6]. Two-step galactokinase-mediated recombineering [7] was used as described previously [8]. In step one, a *galK* cassette encoding galactokinase was inserted into the BAC at the locus to be modified by PCR amplification of plasmid pgalK using the oligonucleotide pairs listed in Table 1, transformation of the PCR product into *E. coli* strain SW102 cells containing the TL12 BAC, colony selection on Gal-positive selection plates, and verification of a clone containing the expected *galK* insertion by PCR and targeted sequencing, as described previously [9]. In step two, synthetic oligonucleotides containing the desired modifications (Table 1) were annealed, and single-stranded overhangs were filled in using PCR. The linear double-stranded DNA product was then transformed into SW102 cells containing the BAC with the *galK* insertion, and colonies were isolated using Gal counterselection plates [7]. Clones containing the desired modifications were then identified using PCR screening and confirmed using targeted Sanger sequencing.

Viruses were reconstituted from BAC DNA by transfection into MRC-5 cells in T25 flasks as described previously [6]. When the flask exhibited extensive infection (based on cytopathic effect and GFP expression), the culture medium was removed, and cells were trypsinized, recombined with the culture medium, and sonicated. Sonicates were clarified by centrifugation at 500× *g* for ten minutes (min.), adjusted to 0.2 M sucrose, and stored in liquid nitrogen. Frozen viral stocks were titered by limiting-dilution using MRC-5 cells in 96-well plates as described previously [10]. Stocks thus derived directly from transfected cultures were designated passage zero (p0). Subsequent passages, performed as above, were designated p1, p2, p4, p6, p10, etc.

### 2.3. Focus Size Determinations

Confluent monolayers of MRC-5 cells in 96-well plates were infected with eight three-fold serial dilutions of virus stocks. After eight days, GFP expression was used to visualize foci using a Nikon TS100 inverted UV fluorescence microscope. ImageJ software was used to calculate the surface areas of GFP-positive foci after manually drawing their perimeters in micrographic images. For each experiment, a minimum of ten foci were measured.

### 2.4. Growth Curves

MRC-5 cell monolayers in 12-well plates were infected at a multiplicity of infection (MOI) of 0.3 plaque forming units (pfu)/cell and maintained with 1.5 mL of culture medium per well. Replicate wells were harvested every other day. The culture medium was removed, and cells were trypsinized, resuspended in 1.5 mL culture medium, and sonicated. Cell sonicates and culture medium were clarified using centrifugation at 500× *g* for ten minutes. Relative levels of infectious virus in culture supernatants and cell sonicates were determined by plating on confluent MRC-5 monolayers in 96-well plates and counting the numbers of GFP+ cells per field of view present on day three post-infection in triplicate wells after imaging with a Nikon TS100 inverted UV fluorescence microscope and 10× objective. 

### 2.5. Immunofluorescence

Infected MRC-5 cultures in chamber slides were fixed in 1% formaldehyde for 30 min. at room temperature, permeabilized with 0.5% Triton X-100 for 20 min. on ice, blocked with 20% FBS in PBS (blocking buffer) for 30 min. at room temperature, then incubated with mouse monoclonal α-FLAG antibody M2 (1:500, Millipore-Sigma, St. Louis, MI, USA) for one hour at room temperature. After washing in blocking buffer, samples were incubated with Alexa Fluor 594-conjugated goat α-mouse antibodies (1:200, Life Technologies, Carlsbad, CA, USA) for one hour at room temperature, followed by nuclear labeling with Hoechst 33342 (0.2 mg/mL, Molecular Probes, Eugene, OR, USA) for three min. at room temperature. For dual staining with α-FLAG and α-pp28 antibodies, MRC-5 cells grown on glass coverslips were strained with a rabbit polyclonal α-FLAG antibody (1:500, Millipore Sigma) and with a mouse monoclonal α-pp28 antibody (1:100, CH19, Virusys Corp., Randallstown, MD, USA) for one hour at room temperature, followed by incubation with Alexa Fluor 594-conjugated goat α-rabbit IgG antibodies and with Alexa Fluor 350-conjugated goat α-mouse IgG (both at 1:200, Life Technologies) for one hour at room temperature. Images were acquired using the 20X objective of a Nikon Eclipse E600 fluorescence microscope equipped with a QImaging Retiga R3 camera. 

### 2.6. Western Immunoblotting

T75 flasks containing confluent MRC-5 cells were infected, and cells were harvested by trypsinization on day five post-infection, lysed in 400 μL Laemmli buffer (Bio-Rad, Hercules, CA, USA), and stored at −20 °C. Aliquots were adjusted to 5% β-mercaptoethanol and heated to 95 °C for five min. prior to separation on 10% Mini-PROTEAN TGX polyacrylamide gels (Bio-Rad) using Tris/glycine/SDS running buffer (Bio-Rad). Proteins were then transferred to a 0.4 μm nitrocellulose membrane (Bio-Rad) using Tris/glycine transfer buffer (Bio-Rad) with 20% methanol. The membrane was blocked for one hour in PBS with 0.02% Tween-20 (Fisher) and 5% powdered milk, then incubated at 4 °C overnight with mouse monoclonal antibodies M2 to FLAG (1:500), MAB810 to CMV IE1 and IE2 proteins (1:500, Fisher Scientific, Hampton, NH, USA), or AC-74 to β-actin (1:2000, Millipore Sigma). After washing with PBS 0.02% Tween-20, the membrane was incubated with a horseradish peroxidase-conjugated goat α-mouse IgG antibody (1:5000, Thermo-Fisher) in blocking buffer for one hour at room temperature, washed with PBS 0.02% Tween-20, and incubated for five min. with enhanced chemiluminescence western blotting substrate (Thermo-Fisher). Chemiluminescence was detected using a ChemiDoc imaging system (Bio-Rad).

### 2.7. Sequencing, Sequence Analyses, Alignments, and Statistical Methods

PCR amplification of affected regions and Sanger sequencing (Eurofins Genomics, Louisville, KY, USA) were used to verify BAC constructs and identify mutations affecting *RL13*. Sequence analyses were performed with the MacVector 18 sequence analysis software package. *RL13* gene sequences were obtained from whole genome sequence files of CMV strains Merlin (NC006273), KG (MN274568), and Towne-varL (GQ121041). A single nucleotide insertion that frame shifts *RL13* in Towne-varL was removed in silico to reconstruct the wild-type Towne *RL13* sequence, and sequences encoding the FLAG epitope (DYKDDDDK) were inserted just before the stop codon. Resulting *RL13* open reading frames were translated into amino acid sequences and aligned using ClustalW. To assess the prevalence of polymorphisms at residue E208, the NCBI database was searched for files containing RL13 sequences using BLASTn. Retrieved sequences were translated and aligned to strain Towne RL13 using ClustalW and then scanned visually for polymorphisms at position 208. GraphPad Prism 5 software was used to graph quantitative data, determine standard deviations, and analyze statistical significance using Student’s two-tailed *t*-test.

## 3. Results

### 3.1. Repair of the RL13 Mutation in TL12 Results in Reduced Focus Size

**Figure 1:**
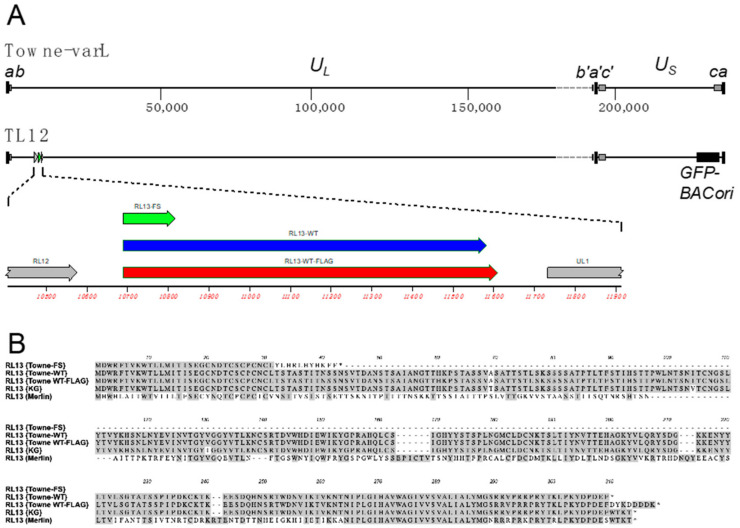
The CMV strain Towne genome and genetic modifications made to *RL13*. (**A**) Top: map of the strain Towne-varL genome showing the *a*, *b*, and *c* sequences and their inverted *a′*, *b′*, and *c′* sequence duplications that flank the unique-long (*U_L_*) and unique-short (*U_S_*) regions. A gray dashed line indicates *U_L_/b′* sequences that are present in Towne-varL but deleted in Towne-varS. Bottom: map of the Towne-varL genome as cloned in BAC TL12 showing the location of *RL13* and of the BAC origin/GFP cassette. Expanded area: *RL13* open reading frames present in the parental virus TL12-RL13^FS^, which contains a one-bp insertion that frame shifts and prematurely truncates RL13, in the TL12-RL13^WT^ virus, where the one-bp insertion has been removed, and in the TL12-RL13^WT-FLAG^ virus, in which sequences encoding a C-terminal FLAG epitope (DYKDDDDK) have been inserted. (**B**) ClustalW alignment of RL13 protein sequences as encoded by viruses TL12-RL13^FS^, TL12-RL13^WT^, and TL12-RL13^WT-FLAG^. RL13 sequences encoded by strains KG and Merlin are included to illustrate that RL13 encoded by TL12-RL13^WT^ is similar to RL13 of KG but highly divergent from RL13 of Merlin.

The Towne strain is derived from the Towne vaccine, which was attenuated by >125 serial passages in WI-38 human fibroblasts [11]. The vaccine is comprised of two genome variants, Towne-varS and Towne-varL (also known as Towne-short and Towne-long). Towne-varL retains the *U_L_/b′* sequences that are deleted from Towne-varS and has short (59-bp) *b/b′* repeats similar to CMV clinical strains (Figure 1A) [12,13,14,15]. Both variants share point mutations disrupting *UL1*, *UL40*, *US1*, *US9*, and *UL130* and a single-nucleotide insertion in *RL13*, resulting in a frame shift (FS) and early termination (Figure 1A,B) [6]. 

All viruses used in this work are derived from BAC clone TL12, which represents the Towne-varL genome (Figure 1A) [6]. The parental virus derived from BAC TL12 is referred to as TL12-RL13^FS^ to differentiate it from viruses in which the *RL13* gene has been modified. In addition, TL12-RL13^FS^ contains a GFP marker cassette within the BAC origin of replication, which is inserted in the *US* region with an accompanying 7.6-kb deletion that truncates *US28* and removes *US29, US30, US31, US32, US34, US34A,* and *TRS1* (Figure 1A) [6]. TL12 replicates efficiently in fibroblasts, produces high titers of cell-free virus [6], and is a highly cell culture-adapted virus. 

**Figure 2:**
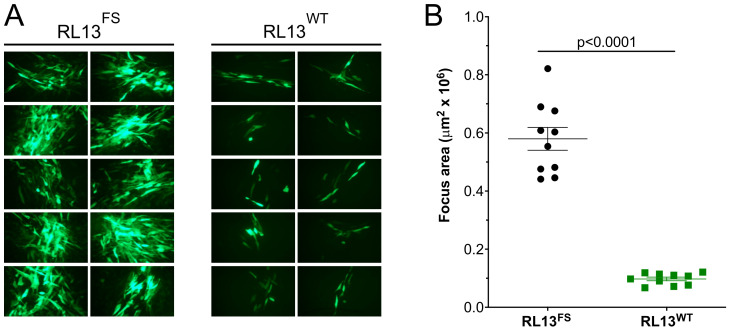
Repair of RL13 reduces focus size in the CMV Towne-varL genetic background. MRC-5 fibroblast monolayers were infected at low MOI with p0 stocks of viruses TL12-RL13^FS^ or TL12-RL13^WT^. (**A**) After eight days, ten GFP+ foci formed by each virus were randomly selected and photographed. (**B**) Areas of GFP+ foci measured using the ImageJ software (*p* value from Student’s two-tailed *t*-test). Original magnification: 100×.

GalK recombineering in *E. coli*, as described in Section 2, was used to repair the *RL13* mutation in the TL12 BAC to the presumed wild-type sequence by removing the one-bp insertion. The resulting *RL13*-repaired BAC was transfected into fibroblasts to produce a p0 stock of virus designated TL12-RL13^WT^. To compare the size of foci produced by TL12-RL13^WT^ to those produced by the parental virus TL12-RL13^FS^, MRC-5 cell monolayers were infected at low MOI, and the size of individual foci produced by each virus was determined based on its GFP+ surface area at day eight post-infection. TL12-RL13^WT^ grew slowly and produced much smaller foci compared to the parental virus TL12-RL13^FS^ (Figure 2A). The surface areas of ten GFP+ foci formed by each virus were measured at day eight post-infection using the ImageJ software. Foci formed by TL12-RL13^WT^ were fivefold smaller than those formed by TL12-RL13^FS^ (*p* < 0.0001) (Figure 2B). Thus, expression of an intact RL13 protein significantly impaired virus growth in the context of the highly cell culture-adapted Towne strain genome. 

### 3.2. RL13 in TL12-RL13^WT^ Mutates after a Few Passages

To determine if the extent of growth restriction imparted by RL13 is sufficient to promote *RL13* gene mutation, the p0 stock TL12-RL13^WT^ was inoculated into replicate flasks of either HFF or MRC-5 fibroblasts, and two lineages were independently passaged on the corresponding cell type. The lineage that was serially passaged on MRC-5 cells led to the appearance of a two-bp deletion frame-shifting *RL13* after codon 28, while the lineage passaged on HFF cells resulted in a five-bp deletion frame-shifting *RL13* after codon 117. The sequence chromatograms (not shown) indicated that these mutations became dominant by passage ten (p10) and passage six (p6), respectively. Thus, expression of an intact RL13 protein was sufficiently restrictive of virus growth to promote the rapid mutation of *RL13* during cell culture passage, suggesting that other adaptive mutations acquired during extensive fibroblast passage of the Towne strain do not mitigate RL13’s temperance activities. 

### 3.3. RL13-FLAG Is Functional

**Figure 3:**
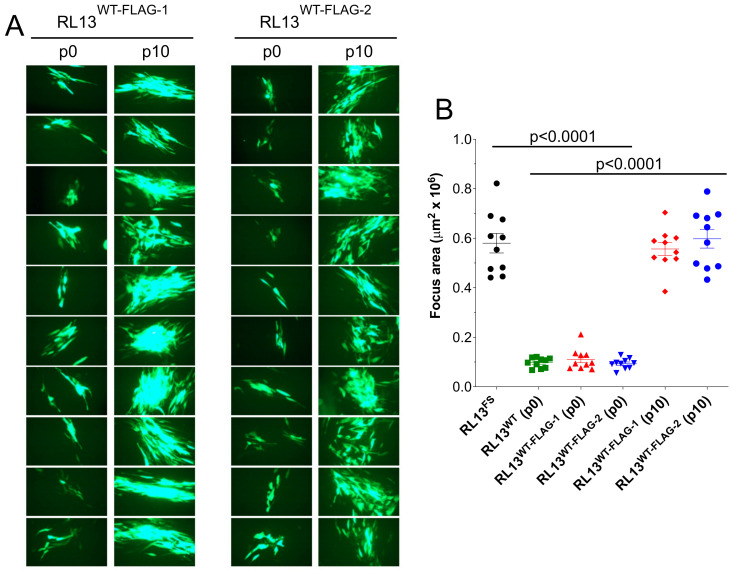
RL13-FLAG retains the ability to restrict focus size. Replicate p0 stocks of TL12-RL13^WT-FLAG^ were passaged as independent lineages, designated TL12-RL13^WT-FLAG–1^ and TL12-RL13^WT-FLAG–2^, then used to infect MRC-5 fibroblast monolayers at low MOI. After eight days, GFP+ foci were photographed (**A**) and their surface areas measured (**B**) as described for Figure 2. Data for viruses TL12-RL13^FS^ and TL12-RL13^WT^ from Figure 2 are included for comparison (*p* values from Student’s two-tailed *t*-test). Original magnification: 100×.

Since antibodies specifically recognizing RL13 have not been reported, a FLAG epitope tag was fused to the C terminus of RL13 in both the TL12-RL13^WT^ and TL12-RL13^FS^ BACs, resulting in viruses TL12-RL13^WT-FLAG^ and TL12-RL13^FS-FLAG^. Independent transfections of TL12-RL13^WT-FLAG^ BAC DNA in MRC-5 cells produced two replicate p0 stocks that were serially passaged as independent lineages designated TL12-RL13^WT-FLAG–1^ and TL12-RL13^WT-FLAG–2^. Foci formed at p0 by TL12-RL13^WT-FLAG–1^ or TL12-RL13^WT-FLAG–2^ were fivefold and sixfold smaller, respectively, than those formed by TL12-RL13^FS^ (*p* < 0.0001) (Figure 3). However, by p10, focus sizes of both TL12-RL13^WT-FLAG–1^ and TL12-RL13^WT-FLAG–2^ lineages had increased to match those of TL12-RL13^FS^ (Figure 3). Targeted PCR sequencing of passage five (p5) stocks identified a predicted E208K substitution in TL12-RL13^WT-FLAG–1^, and a two-bp deletion/frame-shift mutation truncating RL13 after H116 in TL12-RL13^WT-FLAG–2^. Wild-type sequences were not evident in the chromatograms (not shown), indicating that both mutations were dominant by p5. Together, these results indicate that addition of the C-terminal FLAG epitope to RL13 does not impair its temperance activities. 

### 3.4. Detection of RL13^WT-FLAG^ Using Immunofluorescence and Western Blotting 

**Figure 4:**
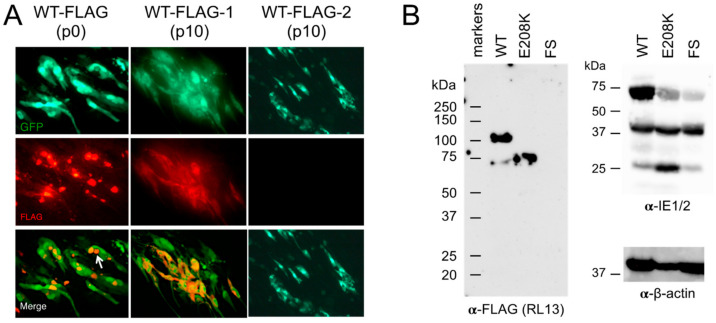
Detection and localization of RL13-FLAG expressed by virus TL12-RL13^WT-FLAG^. (**A**) MRC-5 cultures were infected with a p0 stock of TL12-RL13^WT-FLAG^ or with p10 stocks of TL12-RL13^WT-FLAG–1^ or TL12-RL13^WT-FLAG–2^. At day eight post-infection, cultures were stained for FLAG, and images of the FLAG (red) and GFP (green) signal were acquired. The white arrow indicates RL13^WT-FLAG^ accumulation into juxtanuclear structures corresponding to the VAC. Original magnification: 100×. (**B**) MRC-5 cultures were infected with p0 stocks of TL12-RL13^WT-FLAG^, TL12-RL13^E208K-FLAG^, or TL12-RL13^FS-FLAG^. Cell lysates prepared at day five post-infection were separated by SDS-PAGE, transferred to a nitrocellulose membrane, and probed using α-FLAG, α-IE1/2, or α-β-actin monoclonal antibodies. The positions of protein molecular weight standards are indicated.

MRC-5 monolayers infected with the p0 stock of TL12-RL13^WT-FLAG^ or the p10 stocks of TL12-RL13^WT-FLAG–1^ or TL12-RL13^WT-FLAG–2^ were stained at day eight post-infection with an α-FLAG monoclonal antibody followed by Alexa Fluor 594-conjugated α-mouse secondary antibodies. RL13-FLAG was detected in large perinuclear structures consistent with the VAC in cells infected with the p0 stock of TL12-RL13^WT-FLAG–1^, but it dispersed within the cytoplasm in cells infected with the p10 stock (Figure 4A), suggesting that the E208K substitution predominant in this latter stock was associated with a partial re-localization of RL13 away from the VAC. No FLAG signal was detected in cells infected with the p10 stock of TL12-RL13^WT-FLAG–2^ (Figure 4A), consistent with the dominant presence of a two-bp deletion/frame shift in RL13 eliminating expression of the C-terminal FLAG epitope. Similarly, no signal was detected in cells infected with TL12-RL13^FS-FLAG^. 

To eliminate the possibility of additional mutations elsewhere in the viral genome affecting focus size or RL13 localization, the TL12-RL13^WT-FLAG^ BAC was modified in *E. coli* to TL12-RL13^E208K-FLAG^ BAC, encoding RL13^K208^ with no additional mutations. Protein species with approximate molecular weights (MWs) of 100 and 75 kDa were detected using the α-FLAG antibody in lysates of cells infected with the p0 stock of TL12-RL13^WT-FLAG^, while only the 75-kDa peptide or no signal were detected in lysates of cells infected with the TL12-RL13^E208K-FLAG^ or the TL12-RL13^FS-FLAG^ virus, respectively (Figure 4B). Taken together, these results confirm that RL13-FLAG is expressed during infection and suggest that RL13 requires VAC localization to exert its temperance functions, as non-functional proteins carrying the E208K substitution are mis-localized into the cytoplasm.

### 3.5. The E208K Mutation Disrupts RL13 Localization and Function

**Figure 5:**
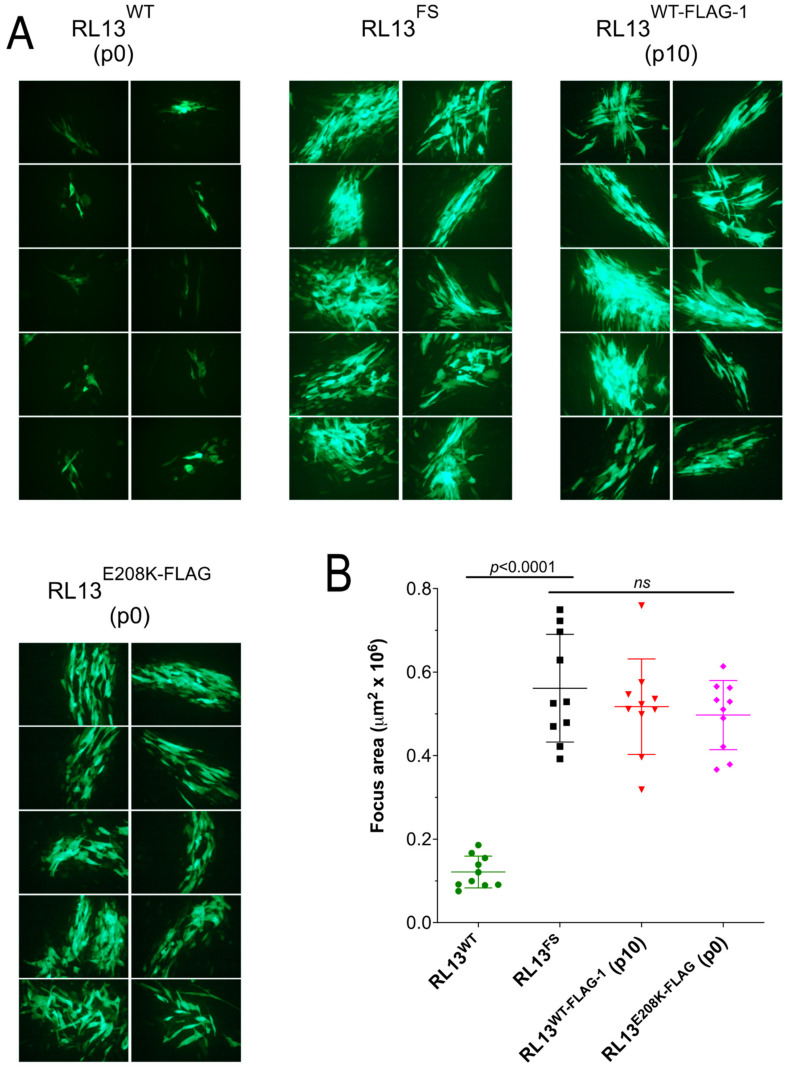
The E208K substitution in RL13 increases focus size. The indicated virus stocks were used to infect MRC-5 fibroblast monolayers at low MOI. After eight days, GFP+ foci were photographed (**A**) and their surface areas measured (**B**) as described for Figure 2 (*p* values from Student’s two-tailed *t*-test; *ns*, not significant.). Original magnification: 100×.

**Figure 6:**
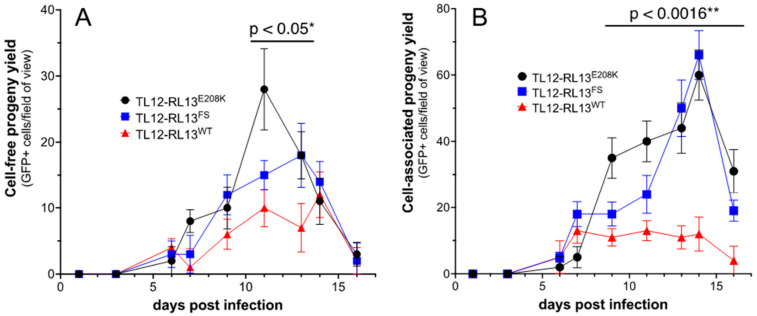
The E208K substitution relieves RL13 restriction of viral replication. Replicate MRC-5 cultures were infected with the indicated viruses at an MOI of 0.3 pfu/cell. On alternate days, post-infection progeny virus yields were determined in culture supernatants (cell-free progeny, **A**) or in cell sonicates (cell-associated, **B**) by infecting fresh monolayer cultures and counting the numbers of GFP+ cells per field of view at day three post-infection. Data shown are means of triplicate assays +/− one standard deviation. * TL12-RL13^E208K^ vs. TL12-RL13^WT^, days 11–13; ** TL12-RL13^E208K^ vs. TL12-RL13^WT^, days 9–16.

A search of 15 available RL13 sequences in the NCBI database revealed that E208 is 100% conserved, suggesting that mutation to K208 may negatively impact RL13 functions. GFP-based analysis of focus size revealed that viruses in the p0 stock of TL12-RL13^E208K-FLAG^ formed large foci similar in size to those of TL12-RL13^FS^ and the p10 stock of TL12-RL13^WT-FLAG–1^ (Figure 5). TL12-RL13^E208K-FLAG^ and TL12-RL13^FS-FLAG^ also exhibited similar levels and kinetics of infectious progeny production, while cell-free and cell-associated TL12-RL13^E208K-FLAG^ progeny production peaked at 2.8-fold and 5.5-fold higher, respectively, than that of TL12-RL13^WT-FLAG^ (Figure 6). 

**Figure 7:**
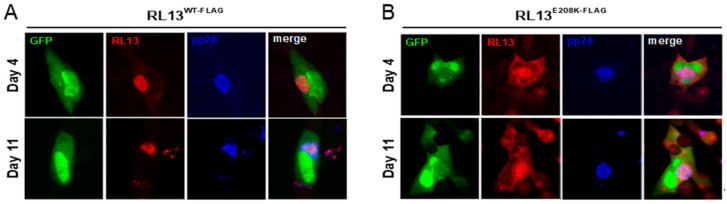
RL13-FLAG localizes to VAC and is mis-localized by the E208K substitution. MRC-5 monolayers were infected with p0 stocks of TL12-RL13^WT-FLAG^ (**A**) or TL12-RL13^E208K-FLAG^ (**B**). After four or 11 days, the cultures were imaged for GFP (green), stained for RL13 with an α-FLAG antibody (red), or stained for pp28 with an α-pp28 antibody (blue). Original magnification: 200×.

Cells infected with TL12-RL13^WT-FLAG^ were then dual stained for RL13 and for pp28, an established VAC marker [16]. RL13-FLAG was again exclusively localized into large, pp28+ juxtanuclear structures (Figure 7A), while RL13-E208K-FLAG remained partially VAC-associated but with substantial additional cytoplasmic localization (Figure 7B). 

### 3.6. Emergence of RL13 Mutations Can Be Quantitated by Immunofluorescence

To determine if the emergence of *RL13* mutations can be monitored using RL13-FLAG localization, HFF cell monolayers were infected with intermediate passages of the TL12-RL13^WT-FLAG–1^ and TL12-RL13^WT-FLAG–2^ virus lineages at low MOI. At day eight post-infection, cells were stained for FLAG, and GFP-positive foci were then scored based on the presence/absence of the FLAG signal or based on RL13-FLAG localization in the VAC only or in the VAC plus the cytoplasm (Table 2). The RL13-FLAG signal was exclusively VAC-associated in all GFP+ foci found in cells infected with p0 stocks. The dispersed/cytoplasmic RL13-FLAG localization pattern associated with the E208K substitution was first observed at p2 of the TL12-RL13^WT-FLAG–1^ lineage, while the FLAG-negative phenotype resulting from the frame-shift mutation was similarly first detected at p2 of the TL12-RL13^WT-FLAG–2^ lineage. In both lineages, the proportion of viruses that gave rise to foci with altered RL13 phenotypes was about 20% at p2, 70–90% at p4, and predominant at p10 (Table 2).

## 4. Discussion

While *RL13* mutations have been observed in the majority of cell culture-passaged CMVs, rare instances in which *RL13* remained intact during extended serial passage suggest the possibility that mutations or polymorphisms affecting genes elsewhere in the CMV genome might mitigate RL13’s negative effects on virus growth [2,17,18,19]. As prior studies of RL13 were conducted predominantly in the context of the genetically authentic strain Merlin or of recent clinical isolates, it was not known whether viruses passaged extensively in cell culture may also have acquired such second-site RL13-mitigating mutations. The present study demonstrates that acquisition of *RL13* mutations reversing the negative pressures imposed by expression of a wild-type version of RL13 are rapidly acquired in the Towne-varL genetic background, which contains numerous adaptive mutations arising during more than 125 fibroblast passages [6,11]. None of these mutations, therefore, appear to act as mitigators of RL13 temperance activities, perhaps because mutations disrupting *RL13* often occur early during passage, eliminating the need for additional mitigating changes. 

In prior studies, Stanton et al. showed that in cells infected with a CMV Merlin strain expressing RL13 fused to a V5 epitope, the RL13-V5 protein co-localized with the cellular trans-Golgi network protein TGN46, the viral tegument protein pp28, and the viral glycoprotein H [2], all established VAC markers [16]. Similarly, we observed exclusive co-localization of Towne RL13-FLAG with pp28 in large perinuclear VACs. By immunoblotting, Stanton et al. observed 100- and 55-kDa species of Merlin RL13-V5 in infected cells, with only the 100-kDa species being present in virions [2]. EndoH and PNGaseF treatments indicated that the disparities in MWs from that of the predicted 35-kDa peptide backbone are due to extensive N- and O-linked glycosylation, and that the 100-kDa species has transited to or beyond the trans-Golgi, consistent with VAC and virion localization [2]. Our immunoblot analysis of Towne RL13-FLAG expressed in cells infected with the TL12-RL13^WT-FLAG^ stock detected a predominant ~100-kDa species and a less abundant ~75 kDa species. Given the extensive sequence divergence between the Towne and Merlin RL13 ectodomains (Figure 1B), these differences in MWs may be due to variations in the prevalence and/or utilization of glycosylation sites. 

Most mutations in *RL13* that arise during serial passage result in profound disruptions, ranging from large deletions removing the entire *RL13* open reading frame, to indels causing frame shifts, to altered start codons, or to premature stop codons [2,20]. Although uncommon, amino acid substitutions have been observed. For example, a T10R substitution in RL13 encoded by strain TB40/E greatly reduces the signal peptide prediction probability [2], suggesting that improper localization may limit RL13’s ability to exert its temperance activities. In prior studies, however, the impact of such substitutions on RL13’s expression, localization, or function have not been further characterized. Using targeted mutagenesis, we verified that the E208K substitution functionally inactivates Towne RL13, resulting in progeny titers and focus sizes comparable to those of the *RL13*-null virus TL12-RL13^FS^. Although the RL13-E208K protein remains at least partially VAC-localized, a significant amount is mis-localized to cytoplasmic regions outside the VAC. Presence of only the 75-kDa species in cells infected by TL12-RL13^E208K-FLAG^ suggests that, perhaps due to mis-localization, RL13-E208K-FLAG is not subjected to the same post-translational modifications that produce the 100-kDa species of RL13-WT. Retention of RL13-E208K-FLAG in the ER, for example, would prelcude specific glycosylation events occurring in the Golgi. Whether mis-localization, altered protein function, or both are responsible for RL13-E208K’s lack of temperance activities remains to be determined.

Because *RL13* can sometimes be eliminated by large deletions [18], detecting the emergence of *RL13*-disruptive mutations using targeted PCR sequencing may be difficult, and while whole genome sequencing can identify and quantitate the presence of large deletions [19], this technique cannot be easily applied to analyze the rate at which *RL13* mutations emerge under different culture conditions, in different cell types, or in the context of strain-specific polymorphisms. In our pilot study, we demonstrate that staining of foci for RL13-FLAG can be used to monitor the emergence of viruses that have lost RL13 expression or exhibit altered RL13 localization during serial passage. Targeted sequencing can then be used to identify the associated mutations. 

Although RL13 is VAC-localized as well as present in the virion envelope [2], the mechanisms by which RL13 limits CMV replication and spread remain unclear. As viral structural proteins coalesce into the VAC to facilitate efficient virion assembly prior to egress [16], RL13 may function to impair or alter virion assembly at this site, or may influence egress pathways to retain virions intracellularly or to redirect them towards cell-to-cell spread. Alternatively, RL13 in released virions may interact with other glycoprotein complexes to modify or impair their roles in virion attachment or entry. It is also possible that RL13 elicits alterations in viral protein expression or DNA replication. Further studies are needed to elucidate the impact of RL13 on each stage of viral replication in order to precisely define its mechanisms of action.

Why RL13 restricts CMV replication is not clear. Stanton et al. speculated that RL13 might alter CMV tropism for certain yet-to-be identified cell types, or it may function as a switch, allowing the timely activation of lytic replication needed for dissemination [2]. Alternatively, de-repression of lytic replication in kidney or salivary glands might increase viral shedding and promote transmission to new hosts. We have also suggested that in the context of a CMV-seropositive host, RL13 may serve to prevent unproductive release of infectious virions into the extracellular space where they would be rapidly inactivated by neutralizing antibodies [18]. Whatever its role, RL13 is clearly important in vivo, as, despite its profoundly negative effects on replication in vitro, CMV genomes with mutations disrupting *RL13* have not been observed in clinical materials [21]. 

## Figures and Tables

**Table 1:** Oligonucleotides used for BAC mutagenesis.

*Modification*	*Step*	*Oligonucleotide*	*Sequence*
RL13^WT^	1	RL13 (TL12)-galk-FW	GATATCTGAAGGTTGCAATGACACGTGCTCCTGTCCGTGCAATTGCCTTTACGACTCACTATAGGGCGAATTGG
		RL13 (TL12)-galk-RV	GCATCGGTGACAGAGTTAGAAGAATTTGTGATAGTGGAGGCGGTGGAGGTGCTATGACCATGATTACGCCAAGC
	2	TL12_RL13 (WT)-FWAN	TAACGTTATGGACTGGAGATTTACGGTTAAGTGGACGTTACTGATGATTACGATATCTGAAGGTTGCAATGACACGTGCTCCTGTCCGTGCAATTGCCTT
		TL12_RL13 (WT)-RVAN	GTTCCATTTGCGATAGCTGAAGTGCTGTTAGCATCGGTGACAGAGTTAGAAGAATTTGTGATAGTGGAGGCGGTGGAGGTAAGGCAATTGCACGGACAGG
RL13^WT-FLAG^	1	RL13 (TL12) FLAG-epitope-galk-FW	GTTATACAAAACTTCCCAAATACGACCCAGATGAATTTTAGACTAAAACCACGACTCACTATAGGGCGAATTGG
		RL13 (TL12) FLAG-epitope-galk-RV	AGACATTATTGGCTAAAAATAAAAACAAAAGTTTATTGATGTGCATGTTAGCTATGACCATGATTACGCCAAGC
	2	RL13 (TL12) FLAG-epitope-FWAN	CGTCCCCAGAAGACCGCGTTATACAAAACTTCCCAAATACGACCCAGATGAATTTTAGACTAAAACCGACTACAAGGACGACGATGACAAGGGACCTAAG
		RL13 (TL12) FLAG-epitope-RVAN	CCACAAAAACCACACGGAGACATTATTGGCTAAAAATAAAAACAAAAGTTTATTGATGTGCATGTTACTTAGGTCCCTTGTCATCGTCGTCCTTGTAGTC
RL13^E208K^	1	E208K-GalK-FW	CCGAACACGCTGGAAAATACGTTTTGCAACGTTACAGTGACGGTAAAAAGGACGACTCACTATAGGGCGAATTGG
		E208K-GalK-RV	GTATAGGAGACGATGTTGCAGTTCCTGATAACACGGTTAAATAGTAGTTTTGCTATGACCATGATTACGCCAAGC
	2	E208K-FWAN	ATATACAACGTAACTACCGAACACGCTGGAAAATACGTTTTGCAACGTTACAGTGACGGTAAAAAGAAAAACTAC
		E208K-RVAN	TACATTTATCAGGTATAGGAGACGATGTTGCAGTTCCTGATAACACGGTTAAATAGTAGTTTTTCTTTTTACCGT

**Table 2:** Emergence of mutations altering RL13 expression or localization.

	*Passage*			
*Lineage*	*0*	*2*	*4*	*10*
RL13-WT-FLAG-1	0/9 ^a^	2/10 ^a^	7/9 ^a^	9/9 ^a^
RL13-WT-FLAG-2	0/10 ^b^	2/10 ^b^	9/10 ^b^	10/10 ^b^

^a^ dispersed/cytoplasmic-FLAG foci/total foci. ^b^ FLAG-negative foci/total foci.

## Data Availability

Not applicable.

## References

[1] Dunn W., Chou C., Li H., Hai R., Patterson D., Stolc V., Zhu H., Liu F. (2003). Functional profiling of a human cytomegalovirus genome. Proc. Natl. Acad. Sci. USA.

[2] Stanton R.J., Baluchova K., Dargan D.J., Cunningham C., Sheehy O., Seirafian S., McSharry B.P., Neale M.L., Davies J.A., Tomasec P. (2010). Reconstruction of the complete human cytomegalovirus genome in a BAC reveals RL13 to be a potent inhibitor of replication. J. Clin. Investig..

[3] Murrell I., Wilkie G.S., Davison A.J., Statkute E., Fielding C.A., Tomasec P., Wilkinson G.W., Stanton R.J. (2016). Genetic Stability of Bacterial Artificial Chromosome-Derived Human Cytomegalovirus during Culture In Vitro. J. Virol..

[4] Dargan D.J., Douglas E., Cunningham C., Jamieson F., Stanton R.J., Baluchova K., McSharry B.P., Tomasec P., Emery V.C., Percivalle E. (2010). Sequential mutations associated with adaptation of human cytomegalovirus to growth in cell culture. J. Gen. Virol..

[5] Cortese M., Calo S., D’Aurizio R., Lilja A., Pacchiani N., Merola M. (2012). Recombinant human cytomegalovirus (HCMV) RL13 binds human immunoglobulin G Fc. PLoS ONE.

[6] Cui X., Adler S.P., Davison A.J., Smith L., Habib S.E., McVoy M.A. (2012). Bacterial artificial chromosome clones of viruses comprising the towne cytomegalovirus vaccine. J. Biomed. Biotechnol..

[7] Warming S., Costantino N., Court D.L., Jenkins N.A., Copeland N.G. (2005). Simple and highly efficient BAC recombineering using galK selection. Nucleic Acids Res..

[8] McVoy M.A., Wang J.B., Dittmer D.P., Bierle C.J., Swanson E.C., Fernandez-Alarcon C., Hernandez-Alvarado N., Zabeli J.C., Schleiss M.R. (2016). Repair of a Mutation Disrupting the Guinea Pig Cytomegalovirus Pentameric Complex Acquired during Fibroblast Passage Restores Pathogenesis in Immune-Suppressed Guinea Pigs and in the Context of Congenital Infection. J. Virol..

[9] Schleiss M.R., Bierle C.J., Swanson E.C., McVoy M.A., Wang J.B., Al-Mahdi Z., Geballe A.P. (2015). Vaccination with a Live Attenuated Cytomegalovirus Devoid of a Protein Kinase R Inhibitory Gene Results in Reduced Maternal Viremia and Improved Pregnancy Outcome in a Guinea Pig Congenital Infection Model. J. Virol..

[10] Cui X., McGregor A., Schleiss M.R., McVoy M.A. (2008). Cloning the complete guinea pig cytomegalovirus genome as an infectious bacterial artificial chromosome with excisable origin of replication. J. Virol. Methods.

[11] Plotkin S.A., Furukawa T., Zygraich N., Huygelen C. (1975). Candidate cytomegalovirus strain for human vaccination. Infect. Immun..

[12] Cha T.A., Tom E., Kemble G.W., Duke G.M., Mocarski E.S., Spaete R.R. (1996). Human cytomegalovirus clinical isolates carry at least 19 genes not found in laboratory strains. J. Virol..

[13] Hahn G., Rose D., Wagner M., Rhiel S., McVoy M.A. (2003). Cloning of the genomes of human cytomegalovirus strains Toledo, TownevarRIT3, and Towne long as BACs and site-directed mutagenesis using a PCR-based technique. Virology.

[14] Prichard M.N., Penfold M.E., Duke G.M., Spaete R.R., Kemble G.W. (2001). A review of genetic differences between limited and extensively passaged human cytomegalovirus strains. Rev. Med. Virol..

[15] Bradley A.J., Lurain N.S., Ghazal P., Trivedi U., Cunningham C., Baluchova K., Gatherer D., Wilkinson G.W., Dargan D.J., Davison A.J. (2009). High-throughput sequence analysis of variants of human cytomegalovirus strains Towne and AD169. J. Gen. Virol..

[16] Das S., Vasanji A., Pellett P.E. (2007). Three-dimensional structure of the human cytomegalovirus cytoplasmic virion assembly complex includes a reoriented secretory apparatus. J. Virol..

[17] Al Qaffas A., Camiolo S., Nichols J., Davison A.J., Ourahmane A., Cui X., Schleiss M.R., Hertel L., Dittmer D.P., McVoy M.A. (2020). Genome Sequence of Human Cytomegalovirus Ig-KG-H2, a Variant of Strain KG Propagated in the Presence of Neutralizing Antibodies. Microbiol. Resour. Announc..

[18] Ourahmane A., Cui X., He L., Catron M., Dittmer D.P., Al Qaffasaa A., Schleiss M.R., Hertel L., McVoy M.A. (2019). Inclusion of Antibodies to Cell Culture Media Preserves the Integrity of Genes Encoding RL13 and the Pentameric Complex Components During Fibroblast Passage of Human Cytomegalovirus. Viruses.

[19] Al Qaffas A., Nichols J., Davison A.J., Ourahmane A., Hertel L., McVoy M.A., Camiolo S. (2021). LoReTTA, a user-friendly tool for assembling viral genomes from PacBio sequence data. Virus Evol..

[20] Dolan A., Cunningham C., Hector R.D., Hassan-Walker A.F., Lee L., Addison C., Dargan D.J., McGeoch D.J., Gatherer D., Emery V.C. (2004). Genetic content of wild-type human cytomegalovirus. J. Gen. Virol..

[21] Suarez N.M., Wilkie G.S., Hage E., Camiolo S., Holton M., Hughes J., Maabar M., Vattipally S.B., Dhingra A., Gompels U.A. (2019). Human Cytomegalovirus Genomes Sequenced Directly from Clinical Material: Variation, Multiple-Strain Infection, Recombination, and Gene Loss. J. Infect Dis.

